# Morphological characteristics of corneal endothelial cells in a healthy southern Han Chinese population across the entire life cycle and evaluation of age-associated endothelial morphological indicators

**DOI:** 10.3389/fmed.2026.1860522

**Published:** 2026-10-08

**Authors:** Jundong Zhu, Qi He, Pu Zhang, Cheng Li, Zhiyuan Li

**Affiliations:** 1 Aier Eye Research Institute, Changsha Aier Eye Hospital, Central South University, Changsha, Hunan, China; 2 Anhui Medical University, Hefei, Anhui, China; 3 The First People’s Hospital of Chenzhou, Hengyang Medical School, South China University, Chenzhou, Hunan, China

**Keywords:** candidate biomarkers, central corneal thickness, endothelial cell density, human corneal endothelium, specular microscopy

## Abstract

**Objective:**

The study aimed to observe the morphological changes in corneal endothelial cells in a healthy Han Chinese population in southern China over the entire life cycle, analyze age-associated morphological changes, and evaluate the potential utility of endothelial assessment parameters as candidate biomarkers.

**Methods:**

In total, 13,820 healthy Han Chinese volunteers were examined by specular microscopy, and the endothelial descriptive indexes, including central corneal thickness (CCT), endothelial cell density (ECD), coefficient of variation in average cell size (CV), percentage of regular hexagonal cells [hexagonality (HEX)], size of the smallest cell (S_min_), size of the largest cell (S_max_), average cell size (S_avg_), and cell area standard deviation (S_sd_), as well as gender and age, were analyzed.

**Results:**

The average age of the participants in this study was 48.14 ± 23.47 (3–99) 14;years. There was no sex predominance, with 6,836 male participants (46.38%) and 6,984 female participants (53.62%). The mean CCT, ECD, CV, HEX, S_min_, S_max_, S_avg_, and S_sd_ were 524.95 ± 34.2 14;μm (415–650 14;μm), 2,843.15 ± 393.84 14;cells/mm2 (1,501–4, 180 14;cells/mm2), 32.07% ± 5.71% (16%–60%), 54.17% ± 12.87% (12%–99%), 146.30 ± 33.81 14;μm2, 790.60 ± 182.74 14;μm2, 357.92 ± 57.92 14;μm2, and 115.71 ± 33.05 14;μm2, respectively. CCT was not significantly associated with age (*p* = 0.061). The cross-sectional age-associated ECD decline was estimated at 0.42% per year of age (95% CI: 0.39%–0.45%). The ECD and hexagonality curves expressed the same linear trend. The CCT and endothelial evaluation indexes had no gender predominance (*p* > 0.05). ECD and HEX were significantly negatively associated with age (*p* = 0.001 or *p* < 0.001). CV, S_min_, S_max_, S_avg_, and S_sd_ were positively correlated with age (*p* < 0.001). Finally, CCT was not significantly associated with age (*p* = 0.061>0.05).

**Conclusions:**

This study found that the ECD decrease rate was 0.42%/year for a healthy Han Chinese population in southern China during the entire life cycle. HEX showed the strongest age-associated sensitivity to morphological changes, while S_max_ and CV demonstrated the most consistent age-related specificity as morphological indicators of endothelial polymegathism and pleomorphism in the specular microscopy results.

## 1 Introduction

The human corneal endothelial cells (hCECs) a monolayer of hexagonal epithelial cells localized on the posterior surface of the cornea, exerts a pivotal regulatory role in maintaining corneal transparency through its well-organized “pump-leak” mechanism (1). This highly organized cellular monolayer actively modulates the transport of water and solutes to preclude corneal stromal edema, while concurrently forming a physiological barrier that restricts the influx of aqueous humor into the corneal stroma (2, 3). In contrast to the majority of other mammalian tissues, hCECs possess extremely limited regenerative potential. Upon injury or functional impairment, hCECs cannot be replenished via intrinsic physiological mechanisms, thereby rendering endothelial cell loss a critical clinical concern in the field of ophthalmology.

Specular microscopy is the primary imaging technique for corneal endothelial evaluation, as it enables non-invasive visualization of endothelial morphology and facilitates quantitative analysis of key parameters, including endothelial cell density (ECD), cell size variation [coefficient of variation (CV)], percentage of hexagonal cells [hexagonality (HEX)], and average cell area (ACA) (1, 2). In clinical practice, comprehensive corneal endothelial assessment is crucial for the maintenance of ocular health, and it is closely associated with the prediction of surgical outcomes and the duration of disease progression.

As a baseline study focusing on the entire life cycle of healthy Han Chinese individuals, this research not only establishes a standard biological sample database but also identifies specific indicators based on common commercial specular microscopy, which may serve as a consistent reference for ocular disease prediction and clinical decision-making.

## 2 Materials and methods

A total of 13,820 healthy Han Chinese volunteers were recruited and enrolled in this cross-sectional study on annual cell loss. After obtaining written informed consent from all participants, each subject underwent a comprehensive ophthalmic examination, including a Snellen visual acuity (VA) test, automatic refraction, slit-lamp biomicroscopy, conjunctival and corneal fluorescein staining, fundus examination, and intraocular pressure (IOP) check. The quantity and phenotype of corneal endothelial cells were evaluated using non-contact specular microscopy (Topcon Noncon specular microscope, SP-1P; Topcon Corporation, Tokyo, Japan) at Changsha Aier Eye Hospital, Central South University, between January 2024 and December 2025. Participants were stratified into 10 age groups: 3–9, 10–19, 20–29, 30–39, 40–49, 50–59, 60–69, 70–79, 80–89, and 90–99 years. Corneal endothelial cell measurements were performed using the autofocus mode, with the central corneal region (4 mm diameter area) selected as the target section for examination and subsequent quantitative analysis. Endothelial descriptive indicators, including central corneal thickness (CCT), ECD, CV, percentage of regular HEX, size of the smallest cell (S_min_), size of the largest cell (S_max_), average cell size (S_avg_), and cell area standard deviation (S_sd_), were collected and subjected to rigorous statistical analysis. Demographic and clinical characteristics of the study subjects, including gender and age, were also recorded.

The eight corneal endothelial parameters were quantitatively analyzed using the built-in software of the Topcon SP-1P specular microscopy system (Figure 1). Endothelial descriptive parameters, including the aforementioned indicators, were collected systematically and subjected to rigorous statistical analysis.

**Figure 1:**
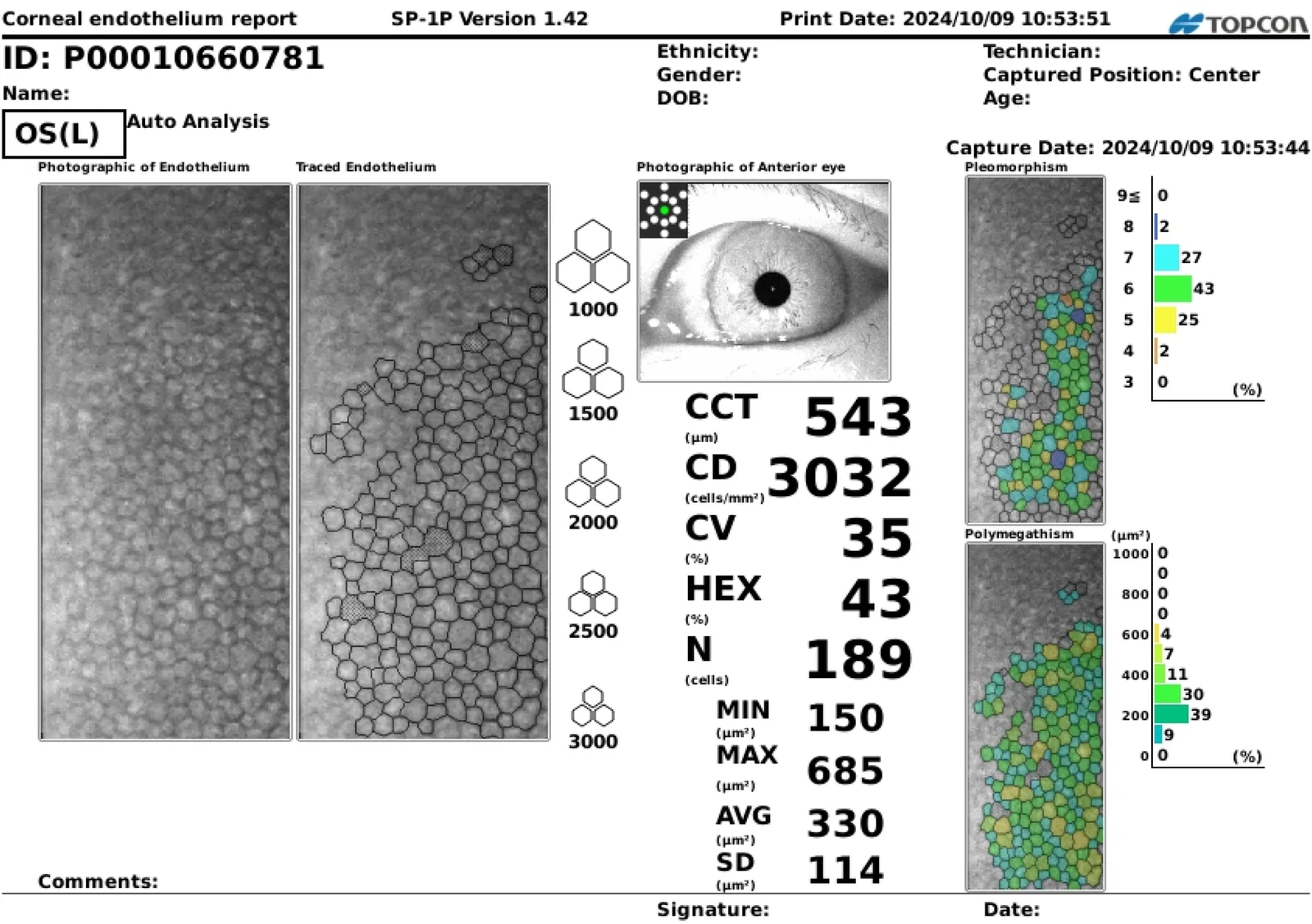
A schematic diagram of human corneal endothelial cell capture using an SP-1P system.

### 2.1 Inclusion and exclusion criteria (4)

The following exclusion criteria were strictly applied to ensure the homogeneity of the study cohort: presence of diabetes mellitus, hypertension, or rheumatoid arthritis; any corneal or intraocular diseases, including keratoconus, keratitis, glaucoma, fundus pathologies such as retinal vein occlusion, central serous chorioretinopathy, age-related macular degeneration (AMD), or use of antiglaucoma medications; corneal disorders (i.e., ectasia, scarring, dystrophy, and dry eye with positive ocular surface fluorescein staining); congenital corneal diseases, congenital iris disorders, and congenital cataracts; pterygium with corneal invasion exceeding 2 mm; recent ocular infection; history of previous ocular surgery or ocular trauma; contact lens wear within 14 days prior to enrollment; and refractive errors with a spherical equivalent beyond ±6 diopters.

Participants were recruited through community health outreach programs conducted in Changsha and surrounding areas of Hunan Province, Southern China, between January 2024 and December 2025. Recruitment methods included public health advertisement postings in community centers, hospitals, and schools; health screening events organized by Changsha Aier Eye Hospital; and voluntary referrals from existing participants. Sampling was consecutive and population-based, with all individuals who met the eligibility criteria during the recruitment period being invited to participate.

A total of 15,236 individuals were initially screened for potential enrollment. Han ethnicity was determined by self-report using the standard ethnic classification system of the People's Republic of China, which recognizes 56 official ethnic groups. All participants self-identified as Han Chinese, the predominant ethnic group in southern China.

Health status was defined based on the following comprehensive assessment.

Systemic health evaluation: Participants underwent a structured medical history interview to exclude the presence of diabetes mellitus (defined as fasting blood glucose ≥126 mg/dL or self-reported diagnosis), hypertension (defined as blood pressure ≥140/90 mmHg or self-reported diagnosis), rheumatoid arthritis, and other significant systemic diseases. Blood pressure was measured using a standard mercury sphygmomanometer after at least 5 min of rest. Fasting blood glucose was measured in a subset of participants (*n* = 3,210) using fingerstick glucometry, and absence of diabetes was confirmed by medical history for all participants.

Ocular health evaluation: Comprehensive ophthalmic examination included Snellen visual acuity testing, automatic refraction, slit-lamp biomicroscopy, conjunctival and corneal fluorescein staining, fundus examination (using indirect ophthalmoscopy or non-mydriatic fundus photography), and IOP measurement using non-contact tonometry. Dry eye was assessed through patient-reported symptoms and positive ocular surface fluorescein staining. Fundus abnormalities (including retinal vein occlusion, central serous chorioretinopathy, and age-related macular degeneration) were excluded by fundus examination and photographic review by retinal specialists.

Of the initial 15,236 individuals, 1,416 were excluded for the following reasons: 412 had incomplete ophthalmic examination data, 356 had systemic diseases (diabetes mellitus, hypertension, or rheumatoid arthritis), 298 had corneal or intraocular diseases, 186 had refractive errors beyond ±6 diopters, 94 had recent contact lens wear within 14 days, and 70 had pterygium with corneal invasion exceeding 2 mm. Thus, 13,820 participants were enrolled, yielding a response rate of 90.7% (13,820/15,236) among eligible screened individuals.

### 2.2 Project design

The ages of the enrolled subjects ranged from 3 to 99 years, and subjects were allocated into 10 groups stratified by age. The volunteers’ distribution is as follows: 3–9 years (567 eyes, 567 patients), 10–19 years (1,320 eyes, 1,320 patients), 20–29 years (1,623 eyes, 1,623 patients), 30–39 years (1,832 eyes, 1,832 patients), 40–49 years (1,698 eyes, 1,698 patients), 50–59 years (1,905 eyes, 1,905 patients), 60–69 years (1,890 eyes, 1,890 patients), 70–79 years (1,672 eyes, 1,672 patients), 80–89 years (894 eyes, 894 patients), and 90–99 years (419 eyes, 419 patients). The number of male and female participants in each group was nearly matched. Only the data from the chosen eye were used to examine the correlations among age, corneal CCT, and endothelial parameters. The human corneal endothelial cell captured model which be uniform under the SOP of equipment.

Sample size estimation was performed using G*Power version 3.1 based on the primary outcome measure (ECD), referencing relevant literature on corneal endothelial parameters in Han Chinese populations. The sample size was calculated using the following parameters: type I error rate (*α*) = 0.05, power (1 − *β*) = 0.95, expected ECD mean ± standard deviation of 2,850 ± 400 cells/mm^2^, and a minimum clinically significant difference in ECD between adjacent age groups of 50 cells/mm^2^. Reference intervals (2.5th–97.5th percentiles) for each corneal endothelial parameter within each age group were estimated using the non-parametric percentile method, as recommended by the Clinical and Laboratory Standards Institute (CLSI) guidelines. The 95% confidence intervals for the reference limits were calculated using the bootstrap method with 10,000 resamples. The minimum required sample size was determined to be 12,564 participants.

The main corneal parameters were calculated, and results were compared among the age groups. Correlations between the main corneal parameters (CCT, ECD, CV, HEX, S_min_, S_max_, S_avg_, and S_sd_) and age were found.

### 2.3 Ethical approval

This study was conducted in strict adherence to the principles of the Declaration of Helsinki, and the study protocol was approved by the Institutional Review Board (IRB) of Changsha Aier Eye Hospital (Approval ID: 2024KYYJ002). All study procedures were performed in accordance with the approved protocol, the Declaration of Helsinki, and relevant clinical research guidelines.

### 2.4 Statistical analysis

Statistical analyses were performed using IBM SPSS® Statistics for Windows, Version 23.0 (SPSS Inc., Chicago, IL, USA). Given that the majority of the data did not follow a normal distribution, non-parametric tests were employed for primary analyses. Student's paired t-test and Pearson's correlation coefficient (*r*) were calculated where appropriate. Analysis of variance (ANOVA) was used to compare the eight corneal endothelial parameters among different study groups. Categorical variables were analyzed using Fisher's exact test or the chi-square test, as appropriate. Multiple linear regression modeling was performed to estimate corneal ECD over time and at different ages. Both bivariate and multivariate analyses were conducted to assess unadjusted and adjusted effects, respectively. All predictors with a *p*-value <0.25 in the bivariate models were considered sufficiently important for inclusion in the multivariate models. An exception was made for ECD, the primary study outcome, which was forced into the multivariate model regardless of its bivariate *p*-value. A two-tailed *p*-value <0.05 was considered statistically significant. We have standardized the reporting of *p*-values throughout: *p*-values <0.001 are reported as “*p* < 0.001,” *p*-values between 0.001 and 0.05 are reported as “ *p* < 0.05,” and *p*-values >0.05 are reported as “no significance”.

## 3 Results

The mean age of participants in this study was 48.14 ± 23.47 years (range, 3–99 years). There was no sex predominance in the study cohort, with 6,836 male (46.38%) and 6,984 female participants (53.62%). The corneal endothelium parameter results in all studied eyes are presented in Table 1. The mean CCT was 524.95 ± 34.29 μm (range, 415–650 μm). The mean ECD was 2,843.15 ± 393.84 cells/mm^2^ (range, 1,501–4, 180 cells/mm^2^). The mean CV was 32.07% ± 5.71% (range, 16%–60%). The mean HEX percentage was 54.17% ± 12.87% (range, 12%–99). The Smin was 146.30 ± 33.81 μm^2^ (range, 23–305 μm^2^). The S_max_ was 790.60 ± 182.74 μm^2^ (range, 417–1,499 μm^2^). The S_avg_ was 357.92 ± 57.92 μm^2^ (range, 229–699 μm^2^). The S_sd_ was 115.71 ± 33.05 μm^2^ (range, 51–299 μm^2^).

**Table 1:** Corneal thickness parameters and endothelial characteristics in healthy Han Chinese participants.

Parameter	Minimal value	Maximal value	Average (mean ± SD)
CCT (μm)	415	650	524.95 ± 34.29
ECD (cells/mm^2^)	1,501	4,180	2,843.15 ± 393.84
CV (%)	16	60	32.07 ± 5.71
HEX (%)	12	99	54.17 ± 12.87
Counted cells (cells)	112	424	194.67 ± 75.48
S_min_ (S/μm^2^)	23	305	146.30 ± 33.81
S_max_ (S/μm^2^)	417	1,499	790.60 ± 182.74
S_avg_ (S/μm^2^)	229	699	357.92 ± 57.92
S_sd_ (S/μm^2^)	51	299	115.71 ± 33.05

CCT, central corneal thickness; ECD, endothelial cell density; CV, coefficient of variability; HEX, hexagonality; S_min_, size of smallest cell; S_max_, size of largest cell; S_avg_, average cell size; S_sd_, size standard deviation.

Table 2 and Figure 2 show the corneal endothelium parameter results in different age groups among healthy Han Chinese participants in South China.

**Table 2:** Corneal thickness parameters and endothelial characteristics in different age groups among healthy Han Chinese participants.

Parameter	Ages 3–9	Ages 10–19	Ages 20–29	Ages 30–39	Ages 40–49	Ages 50–59	Ages 60–69	Ages 70–79	Ages 80–89	Ages 90–99	Hc/F	*p*
Number	567	1,320	1,623	1,832	1,698	1,905	1,890	1,672	894	419		
CCT (μm)	524.69 ± 30.93	524.94 ± 33.01	525.10 ± 32.62	524.99 ± 34.82	524.91 ± 34.10	524.84 ± 33.82	524.86 ± 35.98	525.05 ± 32.77	525.01 ± 40.67	524.97 ± 33.21	0.013	0.061
ECD (cells/mm^2^)	3,358.29 ± 296.86	3,129.90 ± 292.64	2,916.15 ± 311.33	2,853.53 ± 334.9	2,844.39 ± 334.32	2,822.86 ± 320.24	2,778.38 ± 357.14	2,711.86 ± 400.07	2,550.39 ± 426.64	2,442.63 ± 520.8	406.84	<0.001
CV (%)	25.94 ± 3.87	28.04 ± 4.68	29.04 ± 4.05	30.28 ± 4.51	31.92 ± 5.03	32.62 ± 4.67	34.53 ± 4.67	35.57 ± 5.12	36.27 ± 6.16	36.63 ± 7.71	561.26	0.001
HEX (%)	68.07 ± 9.33	62.87 ± 10.48	59.39 ± 9.95	58.98 ± 10.27	54.12 ± 9.16	53.90 ± 9.23	49.08 ± 13.07	45.99 ± 13.68	45.75 ± 12.85	41.80 ± 12.78	526.52	<0.001
Counted cells (n)	236.83 ± 74.85	193.04 ± 71.94	182.89 ± 58.24	202.05 ± 62.31	191.40 ± 65.42	186.46 ± 67.14	202.60 ± 91.60	210.75 ± 90.61	173.80 ± 78.71	151.30 ± 68.56	65.253	<0.001
S_min_ (S/μm^2^)	129.93 ± 22.04	132.50 ± 30.35	139.55 ± 36.24	144.47 ± 27.14	144.50 ± 29.53	148.60 ± 32.53	150.01 ± 28.68	153.43 ± 39.40	157.92 ± 40.49	173.12 ± 40.64	107.27	<0.001
S_max_ (S/μm^2^)	610.88 ± 109.31	660.66 ± 119.83	748.94 ± 170.64	760.34 ± 129.85	774.71 ± 131.24	793.26 ± 171.51	825.26 ± 165.66	873.53 ± 186.51	944.53 ± 218.53	973.54 ± 246.18	395.17	<0.001
S_avg_ (S/μm^2^)	300.39 ± 28.40	322.16 ± 32.04	341.72 ± 36.30	351.96 ± 44.95	357.69 ± 48.03	359.26 ± 45.28	366.55 ± 56.40	377.19 ± 65.15	400.68 ± 74.54	425.03 ± 103.543	336.75	<0.001
S_sd_ (S/μm^2^)	78.92 ± 17.53	91.16 ± 20.27	98.53 ± 18.57	105.79 ± 24.46	114.42 ± 25.6	117.62 ± 24.22	127.41 ± 28.07	135.13 ± 33.24	146.15 ± 39.97	153.99 ± 52.85	665.07	<0.001

CCT, central corneal thickness; ECD, endothelial cell density; CV, coefficient of variability; HEX, hexagonality; S_min_, size of smallest cell; S_max_, size of largest cell; S_avg_, average cell size; S_sd_, size standard deviation; Hc, Kruskal–Wallis H test statistic (used for non-normally distributed parameters); F, one-way ANOVA F statistic (used for normally distributed parameters).

**Figure 2:**
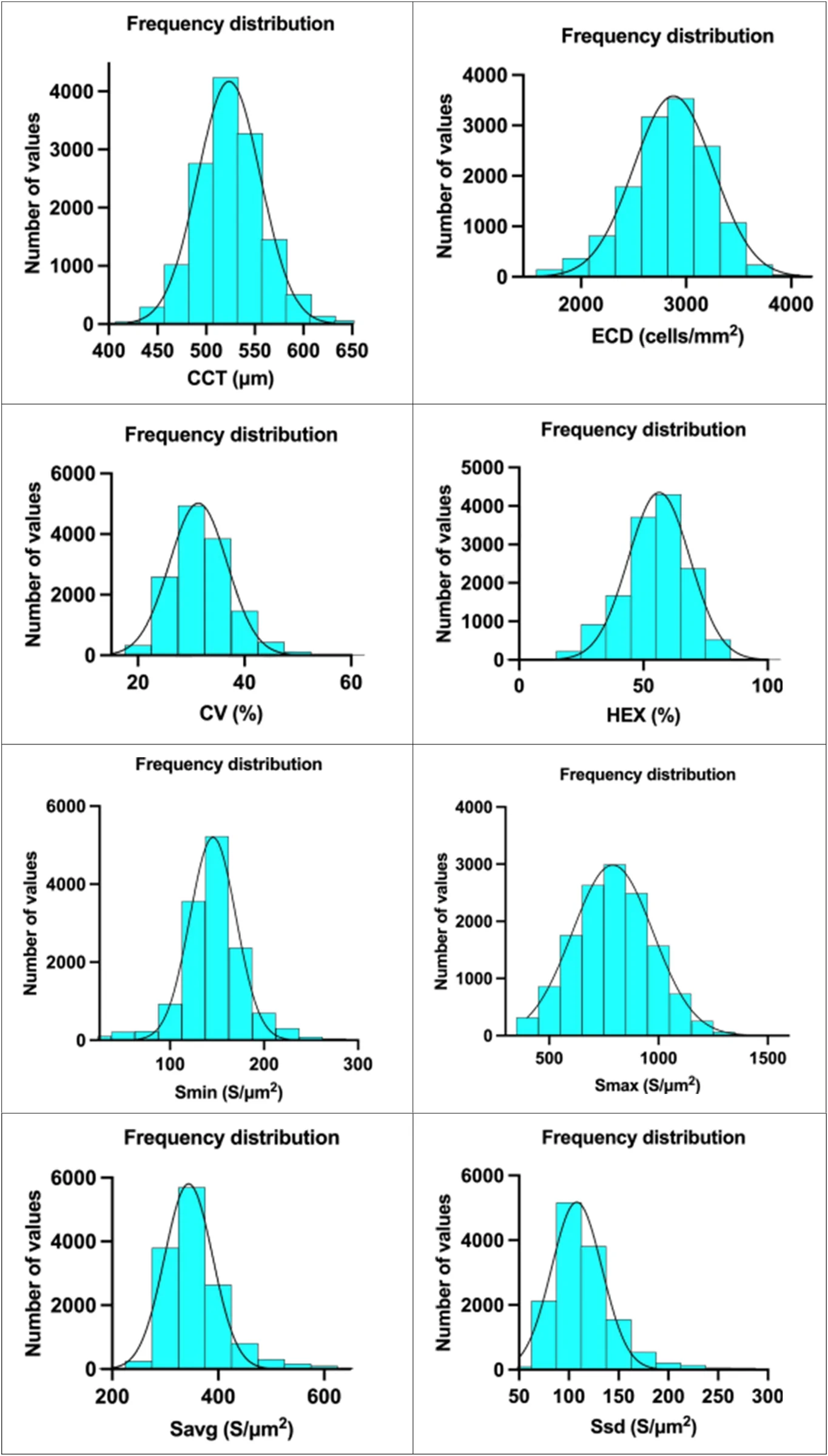
The frequency distribution curve of the endothelial descriptive index. The CCT parameter distribution appears as a stable, normal curve that coincides with the line of S_max_ distribution, although S_max_ is the variable index. CCT can be used as a biomarker for the stability of human corneal endothelial cells across the human lifespan (3–99 years old).

The developmental trends of ECD and HEX were consistent, indicating that both the quantity and functional characteristics of corneal endothelial cells reflect age-related decline. The of CV and HEX curves were complementary: HEX serves as a reliable indicator of the normal physiological status of the corneal endothelium, while CV reflects the phenotypic variability of hexagonal endothelial cells. Although both S_max_ and S_min_ exhibited an increasing trend with age, S_min_ contributes more to the structural integrity and functional stability of the corneal endothelium. Notably, the increase in cell size (both S_max_ and S_min_) was significantly associated with greater fluctuations in corneal endothelial function.

The slope of the weighted regression (*β* = −0.0042, 95% CI: −0.0045 to −0.0039) indicated an estimated decline rate of 0.42% per year, expressed as a percentage relative to the weighted mean ECD across all age groups. This estimate reflects a cross-sectional association and should not be interpreted as a true longitudinal annual loss rate.

The ranges of the eight parameters in the 10 groups are demonstrated in Figure 3 and Figure 4. CCT showed no statistically significant between-group difference (ANOVA, *p* = 0.061>0.05), but the ECD quantity index and the endothelial morphology descriptive indices, namely, HEX, CV, S_min_, S_max_, S_avg_, and S_sd_, demonstrated significant differences according to age (Hc*/*F = 526.52, 561.26, 107.27, 395.17, 336.75, and 665.07, respectively, *p* < 0.001).

**Figure 3:**
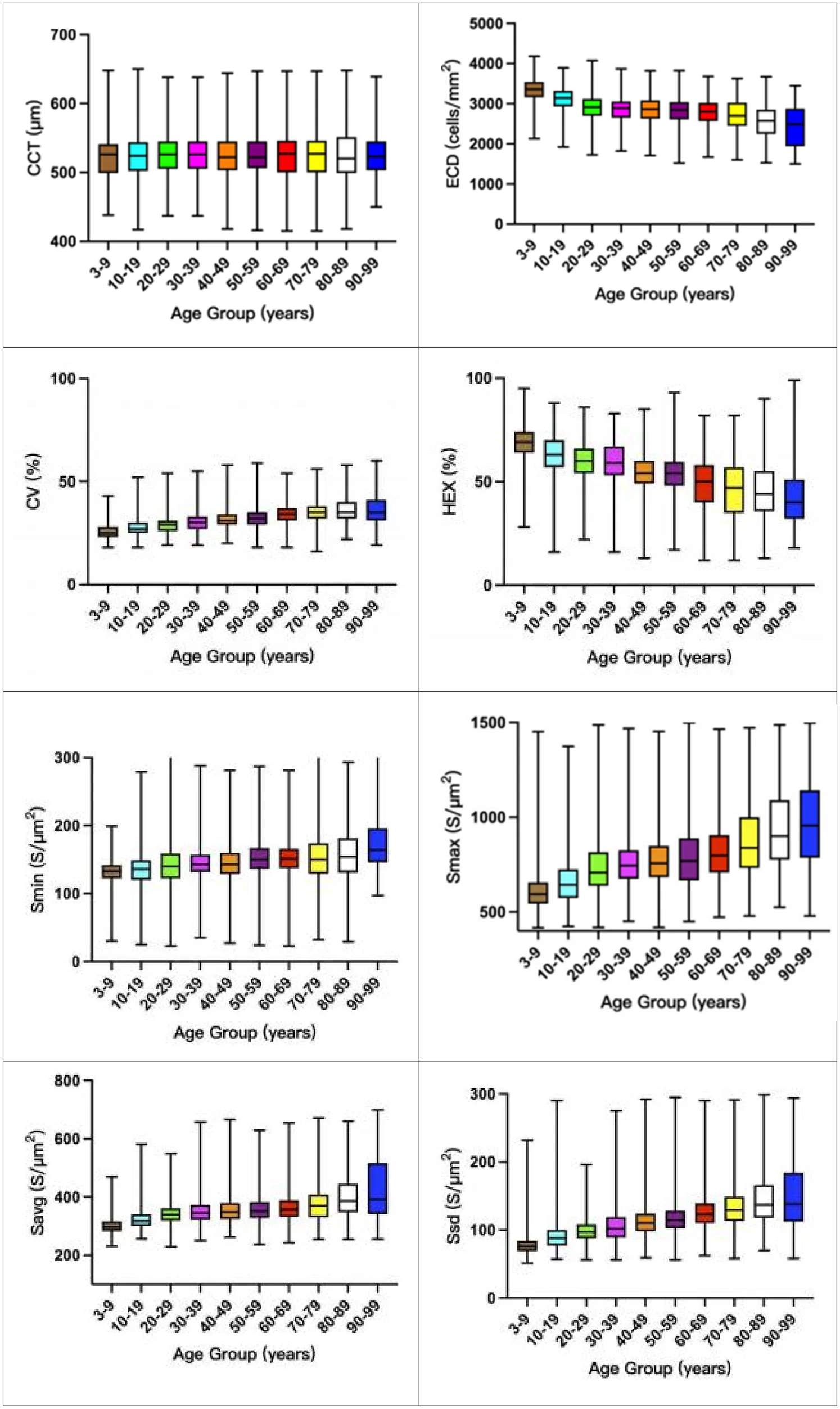
Box plots of the eight endothelial descriptive index parameters in different study groups: **(a)** central corneal thickness, **(b)** endothelial cell density, **(c)** coefficient of variability, **(d)** hexagonality, **(e)** size of smallest cell, **(f)** size of largest cell, **(g)** average cell size, and **(h)** size standard deviation.

**Figure 4:**
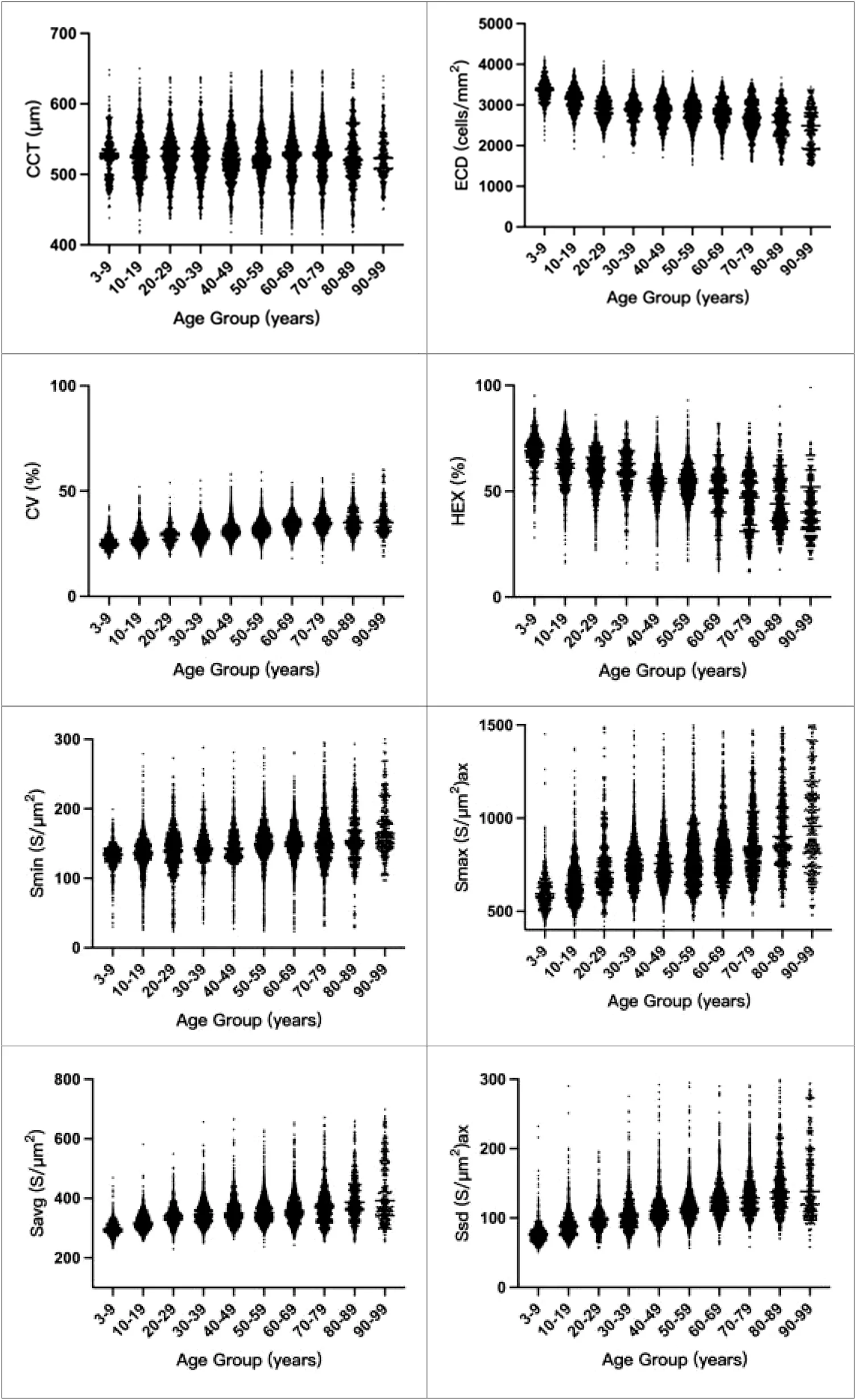
Scatter plots of the eight endothelial descriptive index parameters in different study groups: **(a)** central corneal thickness, **(b)** endothelial cell density, **(c)** coefficient of variation, **(d)** hexagonality, **(e)** size of smallest cell, **(f)** size of largest cell, **(g)** average cell size, and **(h)** Size standard deviation.

Age-stratified reference intervals for each corneal endothelial parameter are presented in Table 3. These reference intervals provide clinically useful benchmarks for the interpretation of individual specular microscopy results across different age groups in this southern Han Chinese population. For example, the expected 2.5–97.5 percentile ranges for ECD were 2,810–3,900 cells/mm^2^ for the 20–29 years group and 1,650–3,350 cells/mm^2^ for the 80–89 years group, illustrating substantial age-associated decline.

**Table 3:** Endothelial cell loss per decade of age.

Age group (years)	ECD (cells/mm^2^) mean ± SD	Upper 95% CI	Lower 95% CI	Cell loss, no (%)
3–9	3,358.29 ± 296.86	3,525.61 ± 311.85	3,190 ± 281.15	
10–19	3,129.90 ± 292.64	3,285.45 ± 306.6	2,972.55 ± 277.92	228 (7.2)
20–29	2,916.15 ± 311.33	3,061.8 ± 326.86	2,770.2 ± 295.73	214 (7.3)
30–39	2,853.53 ± 334.59	2,996.65 ± 351.31	2,710.85 ± 317.86	63 (2.2)
40–49	2,844.39 ± 334.32	2,986.60 ± 350.7	2,701.8 ± 317.6	9 (0.3)
50–59	2,822.86 ± 320.24	2,963.1 ± 336.25	2,168.7 ± 304.2	22 (0.8)
60–69	2,778.38 ± 357.14	2,917.2 ± 374.09	2,722.8 ± 339.2	44 (1.6)
70–79	2,711.86 ± 400.07	2,846.55 ± 420	2,576.26 ± 380	67 (2.5)
80–89	2,550.39 ± 426.64	2,677.5 ± 447.9	2,422.5 ± 424.65	151 (5.9)
90–99	2,442.63 ± 520.78	2,564.7 ± 546	2,329 ± 518.7	108 (4.4)

Cell loss was calculated as the percentage decrease in mean ECD from the previous age group: [(ECD previous − ECD current)/ECD previous] × 100%. This represents a cross-sectional comparison between adjacent age groups, not a longitudinal follow-up of the same individuals. The cross-sectional age-associated ECD decline rate was calculated using a weighted linear regression model with age groups as independent variables and mean ECD as the dependent variable. Age group means were weighted by the inverse of the within-group variance to account for heterogeneity across age groups.

## 4 Discussion

### 4.1 The physiology of human corneal endothelial cells

hCECs are derived from cranial neural crest progenitors, forming a single monolayer of hexagonal cells lining the posterior segments (1). These cells play the unique role of pumping water out of the corneal tissue to maintain corneal stromal dehydration. Otherwise, due to the avascular cornea, hCECs absorb nutrient composites from the anterior chamber into the corneal stroma to maintain cell function and remove edema (2). HCECs play a key role in controlling corneal transparency by manifesting pump function mediated via Na-K-ATPase (3, 4), and barrier function through ZO-1 and peri-junctional actomyosin ring (PAMR) expression (2, 5–7). Moreover, the cornea is the most glycolytic tissue, in which 85% of metabolized glucose is transferred into lactate. As two lactates are produced for each glucose, a potent osmotic pressure is created that must be discharged from the cornea. Lactate-coupled water fluxing is the inner mechanism of the corneal endothelial bio-active pump, which relies on the presence of bicarbonate (8–10).

### 4.2 The fundamental process of endothelial variations

Although human corneal endothelial cells have high metabolic ability, they cannot proliferate *in vivo*, leading to a gradual decrease in cell density at a rate of 0.3%–0.6% per year (11). However, even with this gradual loss, a healthy corneal endothelium normally has an enormous capability to sustain its function throughout an individual's lifetime (2). Ocular surgical trauma (2) and inflammatory disease (12) can result in rapid cell loss. The loss of endothelial cells leads to the enlargement and spreading of the remaining borderline cells over the defective area. As a consequence of this complement mechanism, the remaining cells enlarge in size and vary in shape, commonly defined as polymegathism and pleomorphism, respectively (2). The CV of the mean cell area is a sensitive index of corneal endothelial dysfunction, while hexagonality is a marker of corneal endothelial wound healing (1). Excessive cell loss leads to stromal and epithelial edema, which in turn results in cloudiness and loss of visual acuity.

### 4.3 Endothelial cell density among a healthy Han Chinese population aged 3–99 years

Our cross-sectional data demonstrated an age-associated ECD decline of 0.42% per year of age (95% CI: 0.39%–0.45%) across the lifespan, which is consistent with the range (0.3%–0.6% per year) reported in previous cross-sectional and longitudinal studies (2, 11, 13). Notably, the age-associated decline was not uniform across decades: the steepest decline was observed between the 20–29-year and 30–39-year groups (7.3%), while ECD remained relatively stable between 40 and 59 years old (0.3%–0.8% per decade). An accelerated decline was again observed after 80 years of age (5.9% per decade). However, we emphasize that these estimates are derived from cross-sectional comparisons of different individuals, and therefore cannot be interpreted as true longitudinal loss rates. Cohort effects, survival bias, and secular trends may influence these cross-sectional observations. Although the rate of decline was 5.9% among those 80–89 years old, which was the third highest, the slope in the 90–99-year group remained stable. Thus, the findings of the present study support the existence of interage differences in mean ECD. The mean ECD of 2,843.15 ± 393.84 cells/mm^2^ in our southern Han Chinese population is consistent with previous reports from Chinese populations. Xu Y et al. reported a mean ECD of 2,850 ± 420 cells/mm^2^ in a similar Chinese cohort, while other Asian studies have reported mean ECD values ranging from 2,700 to 2,900 cells/mm^2^ (14, 21). Our values are somewhat higher than those reported in some Caucasian populations (2,400–2,600 cells/mm^2^) (22, 23) and Japanese populations (2,650–2,800 cells/mm^2^) (20), supporting the existence of interracial differences in mean ECD that have been previously suggested (24).

Regarding age-associated decline, our cross-sectional estimate of 0.42% per year falls within the previously reported range of 0.3%–0.6% per year from both cross-sectional and longitudinal studies (2, 11, 15). The pattern of accelerated decline in younger adulthood (20–39 years) and older age (≥80 years) has been observed in several other large cohort studies (16, 17), suggesting a non-linear trajectory of endothelial aging that may reflect both developmental and senescence-related processes.

The age-stratified reference intervals presented in our study contribute to the growing body of normative data for corneal endothelium parameters in Asian populations. Comparisons with studies from Northern China (18) and other Asian regions (19, 20) will be important to determine whether regional variations exist within the broader Chinese population. There is currently no consensus on the best percentile cutoffs for reference limits. Some previous studies have argued that the 3–99 and 5–95 percentiles can be used as cutoffs for reference (16). The most common way to establish reference limits for biological measures in a healthy population has been to consider the central 95% of the reference population (17). This reasoning also holds for ECD.

### 4.4 Gender differences in cell size and morphology

The size and morphology of endothelial cells area unique feature of human development (18). Xie et al.'s study reported that the sharpness of human endothelial cells is associated with age, with cells in childhood obesity found to be significantly different from those in adolescence and early adulthood (19). Aside from age-matched division, our data show that the size of the largest hexagonal or atypical cells was larger among the female participants than the male participants (t = 4.15, *p* = 0.042 < 0.05), which leads to endothelial dysfunction, as women's inner microenvironment may fiuctuate due to hormone or intestinal fiora issues. Ono et al. (20) observed larger endothelial cell size in female subjects compared to males in a Japanese population. However, the underlying mechanisms of this sex difference—whether hormonal, genetic, or environmental—remain unclear and require further investigation. Our study did not directly evaluate endocrine or microbiome factors, and such explanations remain speculative at this stage.

### 4.5 The standard approved proposal for endothelial estimation

Imaging devices that can enable visualization and precise assessment of corneal layers, particularly the corneal endothelium, are crucial. Advances in technology have led to the development of anterior segment imaging modalities that can be useful for monitoring endothelial cells, such as anterior segment optical coherence tomography (AS-OCT), Scheimpflug corneal tomography (21), specular microscopy, and *in vitro* confocal microscopy (22). Moreover, there have been advances in image processing technology and artificial intelligence (AI) (23).

The quantitative indicators ECD and HEX had a significant negative association with age in the multivariate analysis (*p* < 0.001). The average number of corneal endothelial cells decreases with age growth during the whole life cycle. Moreover, the corneal endothelial variability indices, i.e., CV, S_min_, S_max_, S_avg_, and S_sd_, showed positive correlations with age (*p* < 0.001), which means that the uniform phenotype could be polymorphic or irregular cell according to age growth.

### 4.6 The value of HEX and S_max_

Changes in CV and HEX are considered early signs that precede a decrease in ECD (24). A high CV value implies a stressed or traumatized endothelium (25). The regressive CV equation is Y = 1.224 × x + 25.39, but the HEX equation is Y = −2.719 × x + 68.76, which means that although endothelial cells can respond to age or inner changes actively through an enlarged or atypical phenotype, the death rate of classic functional HEX endothelial cells does not change due to age. The regression equation of S_max_ is Y = 33.87 × x + 608.8, which means that the key variation aspect of them is the original “larger” ones, which are much more fluacture with the age growth. These findings coincide with the classic theory that the healthier or more stable endothelial cell is a hexagonal phenotype.

### 4.7 Limitations

First, the cross-sectional design of this study represents a significant limitation in interpreting the age-associated ECD decline. The 0.42% per year estimate is derived from comparisons between different individuals across age groups, not from longitudinal follow-up of the same individuals. Consequently, our findings represent age-associated associations that may be influenced by birth-cohort effects, survival bias, secular trends in health status, environmental exposures, and other unmeasured confounding factors. The observed pattern of ECD decline across age decades does not necessarily reflect the true trajectory of individual-level endothelial aging. Second, this cross-sectional study targeting healthy individuals is likely susceptible to survivor bias, as healthier individuals may be over-represented in older age groups, potentially underestimating the true age-related ECD decline. In addition, the healthy volunteer effect may introduce selection bias, as participants who volunteer for health screenings may differ systematically from the general population in terms of health awareness, socioeconomic status, and health behaviors. Third, the objective data included in this study were obtained using a single modality (specular microscopy). Future studies should consider corroboration with two or more monitoring methods, such as real-time corneal confocal microscopy or high-definition anterior segment tomography, to determine the consistency of the research conclusions. Fourth, the clinical significance of the corneal endothelial CV and S_max_ indicators—observed in this large sample—requires further exploration through big data artificial intelligence approaches, *in vitro* 3D models, or organoid simulations to investigate their mechanisms and predict their impact on corneal and ocular surface diseases. Fifth, measurement-related variability should be considered in interpreting our results. Although we used a single specular microscopy platform (Topcon SP-1P) operated by trained technicians, intra-observer and inter-observer variability in cell counting and parameter estimation may introduce measurement error. The use of autofocus mode and built-in software analysis reduced operator-dependent variability, but we did not formally assess repeatability or reproducibility in this study. Future studies should include independent verification by two or more observers or automated analysis methods.

## 5 Conclusion

The cross-sectional age-associated ECD decline estimate was 0.42% per year of age (95% CI: 0.39%–0.45%) in this healthy southern Han Chinese population across the lifespan. HEX demonstrated the strongest negative association with age, serving as a sensitive morphological indicator of endothelial aging; S_max_ and CV showed consistent positive associations with age, suggesting their utility as specific indicators of endothelial polymegathism. These candidate biomarkers require prospective validation in diagnostic and prognostic settings.

## Data Availability

The original contributions presented in the study are included in the article/Supplementary Material; further inquiries can be directed to the corresponding author.
